# L5 Osteoid Osteoma Treated with Partial Laminectomy and Cement Augmentation

**DOI:** 10.7759/cureus.4239

**Published:** 2019-03-12

**Authors:** J. Manuel Sarmiento, Julie L Chan, Justin D Cohen, Debraj Mukherjee, Ray M Chu

**Affiliations:** 1 Neurosurgery, Cedars-Sinai Medical Center, Los Angeles, USA

**Keywords:** spinal osteoid osteoma, partial laminectomy, cement augmentation

## Abstract

Osteoid osteoma is a benign primary bone tumor of unknown etiology that occurs most commonly in males during adolescence and early adulthood. Osteoid osteoma affects the spine in 20% of cases, and may cause spinal deformity, stiffness, and pain that may sometimes be worst at night. We present a novel description of a partial laminectomy with cement augmentation after resection of an osteoid osteoma.

A 22-year-old male with a past medical history of Hodgkin's lymphoma status post chemotherapy and radiation to the mediastinum, and right hip osteoblastoma treated with surgery and radiofrequency ablation presented with low back pain for five years with a recent onset of severe radicular symptoms. The pain was described as shooting and radiating laterally down the right leg to the mid-calf without bowel or bladder incontinence. He has a known right L5 laminar sclerotic lesion measuring 11 x 10 mm causing neuroforaminal narrowing and it kept increasing in size despite previous treatment with stereotactic radiosurgery and radiofrequency ablation. This lesion was metabolically active on positron emission tomography-computed tomography (PET-CT) imaging. His pain was worsening and was refractory to physical therapy, non-steroidal anti-inflammatory drugs (NSAIDs), aspirin, and radiation therapy.

A right L5 partial laminectomy was performed to resect the abnormality in an en-bloc fashion. The lesion did not involve the inner cortex of the bone. Lamina reconstruction was achieved with bone cement augmentation for the preservation of vertebral column strength. Pathology was consistent with osteoid osteoma with marrow edema. Microscopic findings include bony trabeculae associated with prominent rimming and hypercellular fibroblastic stroma. No nuclear atypia, necrosis or appreciable mitotic activity was observed. The patient remains neurologically intact with significantly improved radicular symptoms and low back pain.

Osteoid osteoma of the lamina may be resected using a partial laminectomy and cement augmentation done to preserve the integrity of the posterior ligamentous complex, prevent potential fracture of the pars interarticularis, and avoid the need for lumbar fusion in younger patients in whom this pathology is commonly found.

## Introduction

Osteoid osteoma is a benign primary bone neoplasm that predominantly affects males in adolescence and early adulthood. Up to 20% of all osteoid osteomas are found in the spine, of which 60% are located in the lumbar spine, 27% in the cervical spine, 12% in the thoracic spine, and 2% in the sacrum [1]. The most common presenting symptom is back pain that may be nocturnal and is relieved with non-steroidal anti-inflammatory drugs (NSAIDs) and aspirin. Surgery is usually curative but the procedure may destabilize the spine and necessitate fusion with instrumentation. Spinal fusion is associated with long-term risks such as heterotopic ossification and development of adjacent segment disease above and below the level of fusion. We present a case of a young adult with spinal osteoid osteoma that was treated with partial laminectomy and cement augmentation to preserve spinal stability, prevent pontential fracture of the pars interarticularis, and obviate the need for spinal fusion.

## Case presentation

Presenting symptoms and workup

**Figure 1:**
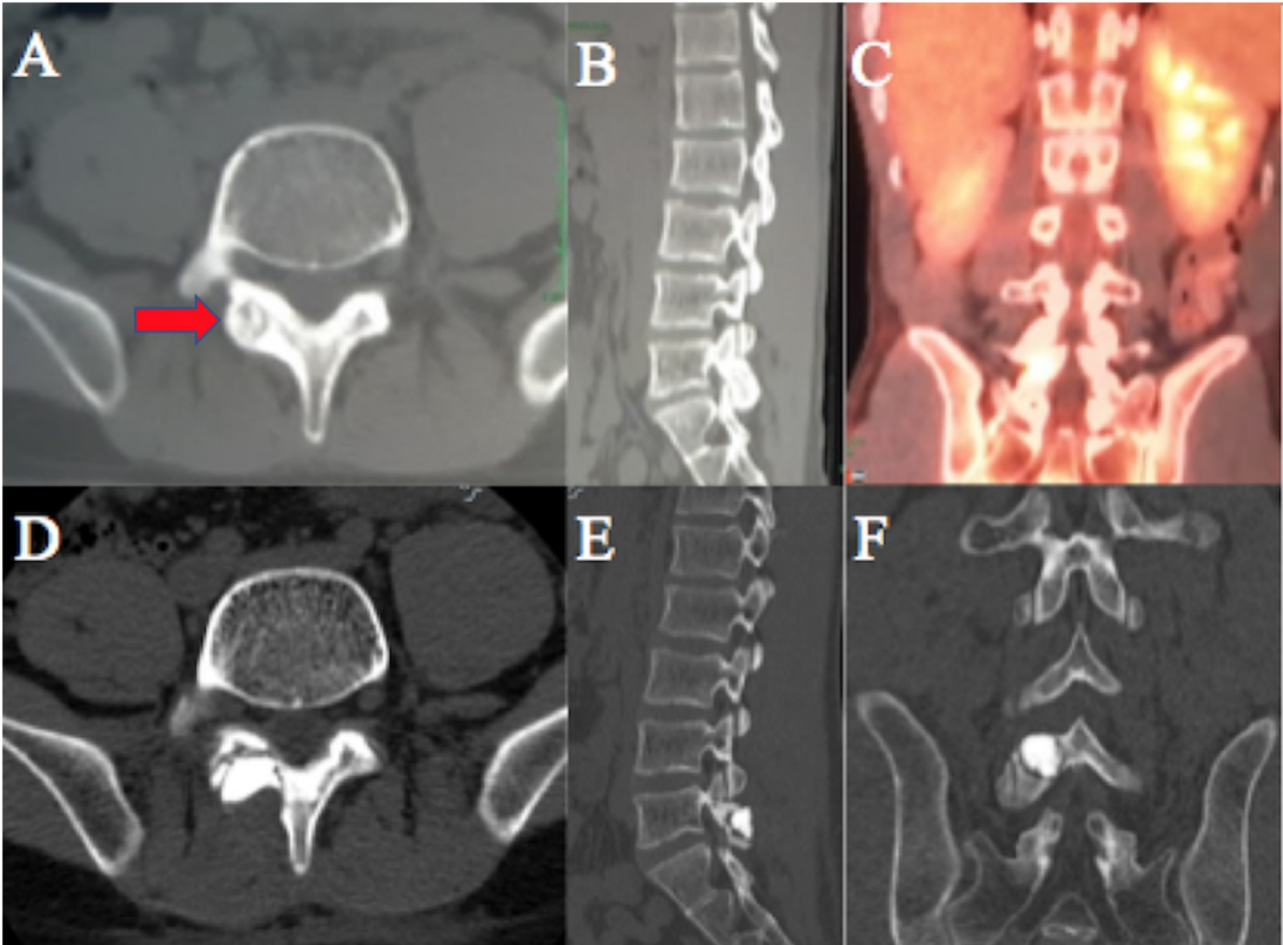
Pre-operative computed tomography (CT) imaging (A) pre-op axial CT showing an 11 x 10 mm sclerotic lesion in the right L5 lamina (red arrow); (B) right L5-S1 foraminal narrowing due to the laminar lesion; (C) positron emission tomography-computed tomography (PET-CT) imaging showing a metabolically active right L5 lamina; (D,E,F) postop axial, sagittal, and coronal CT scans, respectively, after resection of osteoid osteoma with cement augmentation.

**Figure 2:**
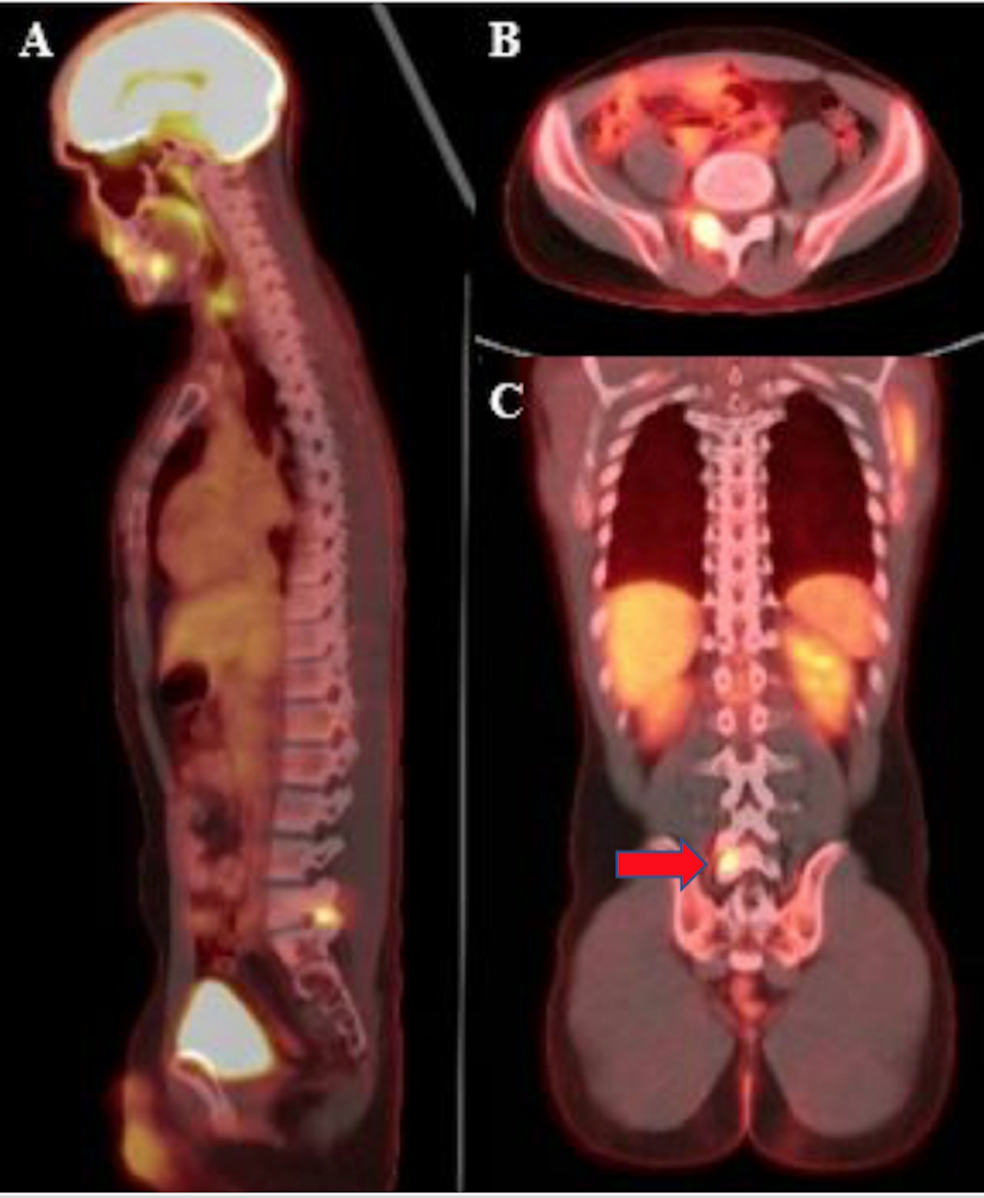
Positron emission tomography-computed tomography (PET-CT) scan (A,B,C) Pre-operative PET-CT scan showing a metabolically active lesion in the right L5 lamina (red arrow) in the sagittal, axial, and coronal planes, respectively.

A 22-year-old male presented with low back pain for five years with a recent onset of severe radicular symptoms. His past medical history was significant for Hodgkin's lymphoma diagnosed in December 2014 that was treated with chemotherapy and radiation to the mediastinum and right hip, as well as osteoblastoma of the right hip treated with surgery and radiofrequency ablation in July 2010. The pain was described as originating from the low back and radiating around his right hip and laterally down the right leg to mid-calf with a shooting-like quality. There was no associated numbness nor bowel or bladder incontinence. He has a known right L5 laminar sclerotic lesion measuring 11 x 10 mm causing neuroforaminal narrowing and increasing in size despite previous treatment with stereotactic radiosurgery and radiofrequency ablation in October 2016 (Figure 1). This lesion encroached near the pars interarticularis and was metabolically active on positron emission tomography-computed tomography (PET-CT) imaging (Figure 2). He reported worsening pain that was refractory to physical therapy, NSAIDs, aspirin, muscle relaxants, lidocaine patches, and radiation therapy. The patient is allergic to sulfa drugs and has a non-contributory social history. Pertinent family history involved his father who died from non-Hodgkin’s lymphoma at the age of 52. On physical examination, the patient had full strength in all extremities including bilateral iliopsoas, gluteals, quadriceps, hamstrings, tibialis anterior, extensor hallucis longus, and gastrocnemius. All reflexes were within normal limits and there were no signs of upper motor neuron disease or muscle atrophy.

Surgical approach

The patient was positioned prone on a Jackson table with padding under all pressure points. A midline lumbar incision was made and a right-sided dissection through the lumbosacral fascia was performed in a subperiosteal fashion. An X-ray confirmed the appropriate level. The right L5 lamina was expanded and raised from the tumor. A partial laminectomy was performed using a high-speed drill to resect the abnormality in an en-bloc fashion, while being careful not to violate the pars interarticularis. The lesion did not involve the inner cortex of the bone. Pieces of Surgicel were used to create a barrier between the cement and the spinal canal. Lamina reconstruction was achieved with bone cement augmentation for preservation of vertebral column strength and prevention of potential fracture of the pars interarticularis.

Pathology

**Figure 3:**
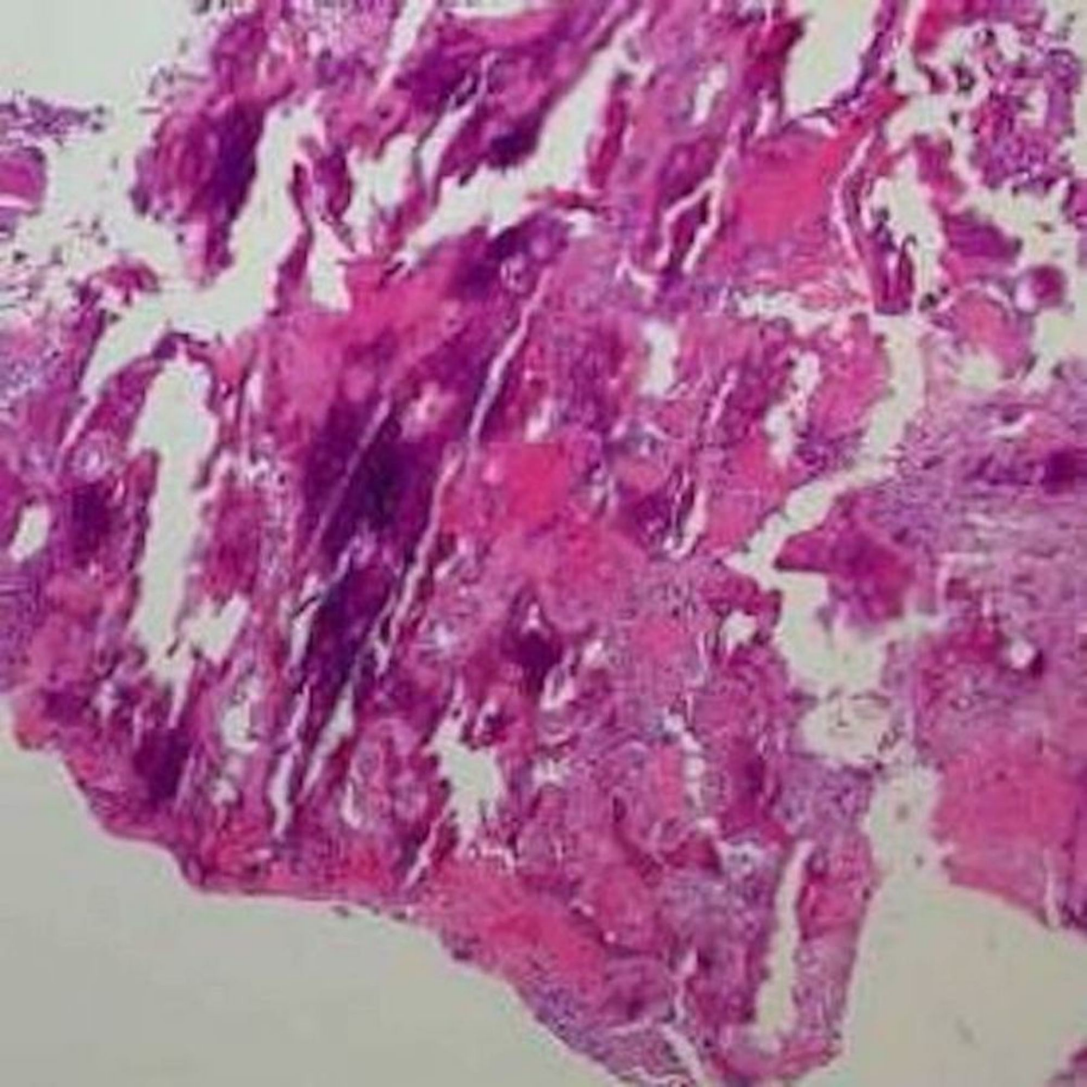
Hematoxylin and eosin (H&E) stain of osteoid osteoma of the spine There is bony trabeculae with prominent rimming and hypercellular fibroblastic stroma. No nuclear atypia, necrosis or mitotic activity is seen.

The pathology was consistent with osteoid osteoma with marrow edema and areas of bone remodeling. No granulomas or evidence of Hodgkin’s disease was identified. Microscopic findings include bony trabeculae associated with prominent rimming and hypercellular fibroblastic stroma (Figure 3). No nuclear atypia, necrosis or appreciable mitotic activity was observed.

Postoperative course

The patient remains neurologically intact with significantly improved radicular symptoms and low back pain. He has decreased his pain medication need and reports improved ambulation 1.5 weeks following surgery.

## Discussion

Osteoid osteomas are rare, primary bone neoplasms comprised of osteoblasts that produce osteoid and woven bone [2]. They are frequently located on long bones and were first described by Dr. Jaffe in 1935 [3]. Osteoid osteomas are smaller, benign and self-limited compared to osteoblastomas, which tend to be larger, more aggressive neoplasms that can undergo malignant transformation [4]. Osteoid osteomas most commonly present in patients during adolescence or early adulthood [5]. There is a male predominance with osteoid osteomas of 2:1-4:1 [6]. Approximately 20% of osteoid osteomas occur in the spine [6]. The thoracolumbar spine is the most common location and they most commonly involve the posterior column of the spine and affect structures such as the facet joints, lamina, pedicles, transverse, and spinous processes [1]. The most common presenting symptom is pain. In fact, the most common cause of painful scoliosis in adolescents is osteoid osteoma of the spine and this important feature separates it from idiopathic juvenile scoliosis-the most common painless cause of scoliosis in childhood [7]. The pain from osteoid osteoma can be nocturnal, occur with activity, and respond to NSAIDs or aspirin (14%-90% of patients have pain alleviated by aspirin) [5].

The differential diagnosis for spinal osteoid osteoma include osteoblastoma, osteosarcoma, aneurysmal bone cyst, Ewing’s sarcoma, and cartilaginous tumors such as osteochondroma and chondrosarcoma. In a patient suspected of having an infection, it is important to consider osteomyelitis. Spinal metastasis must be considered in older patients with axial back pain, neurological deficits, and previous oncologic history. A polyostotic pattern of bone involvement should raise concerns for fibrous dysplasia. Most osteoid osteomas are osteosclerotic on plain radiographs and may not always have a visible nidus. Computed tomography (CT) is most helpful in preoperative planning to determine the exact location and delineate the extent of bony involvement. Magnetic resonance imaging (MRI) is useful to visualize the spinal cord or nerve root compression and extraosseous soft tissue involvement [8]. The vascular nidus may enhance avidly after gadolinium administration [5]. Technetium bone scan is the most sensitive tool in the diagnosis of osteoid osteoma by demonstrating intense focal radionuclide uptake at the lesion site. PET imaging may be helpful in the diagnostic and post-therapy settings. Imperiale et al. showed that the tumor nidus exhibits fluorine-18 fluorodeoxyglucose (18F-FDG)-avid glucose metabolism and metabolically inactive surrounding sclerosis [9]. The nidus was no longer hypermetabolic a month following percutaneous radiofrequency ablation of this lesion.

The medical management of pain from osteoid osteoma consists of NSAIDs or aspirin. The indications for surgery include medically-refractory pain, spinal deformity, evidence of tumor growth, diagnosis, and oncologic cure. The nidus has a red punctiform appearance that is easily distinguished from the surrounding white bone. At surgery, the nidus is generally removed with a curet and then the surrounding reactive bone is gradually excised. Complete resection is achieved in >90% of cases [7-8]. Posterior instrumentation and/or posterolateral fusion is performed when spinal instability occurs as a result of tumor destruction or surgical resection of important weight-bearing posterior elements such as facet joints, pars interarticularis, and pedicles. Most surgeons report a 20%-50% fusion rate in their series of patients with spinal osteoid osteomas [2,5,7-8,10]. The recurrent rate of osteoid osteoma is 4.5% [6]. Radiation is reserved for patients who have an incomplete resection or progressive disease due to the potential risk for the development of post-radiation sarcoma [5].

We present a case of a 22-year-old male surgically cured from osteoid osteoma with a partial laminectomy and cement augmentation in order to preserve spinal stability and avoid unnecessary spinal fusion. A partial laminectomy provides targeted resection of the bony lesion while preserving the integrity of the posterior ligamentous complex that is removed with a standard laminectomy procedure. Cement augmentation was used to reconstruct the remaining lamina and preserve the stability of the spine, obviating the need for extensive spinal fusion and instrumentation. Due to the tumor’s proximity to the pars interarticularis, cement augmentation, in this case, may also serve to avoid a fracture in the pars interarticularis and prevent potential subsequent axial back pain symptoms and spinal instability. Avoiding spinal fusion reduces the risk of excessive blood loss, increased postoperative pain, and protracted hospital length of stay. The patient will also have preserved spinal mobility and have no risk of developing heterotopic ossification and adjacent segment disease later in life.

## Conclusions

Osteoid osteoma of the lamina may be safely resected using a partial laminectomy and cement augmentation to preserve the integrity of the posterior ligamentous complex, prevent potential fracture of the pars interarticularis, and avoid the need for lumbar fusion in younger patients in whom this pathology is commonly found.
